# Externe wissenschaftliche Evaluation der ersten Teledermatologie-App ohne direkten Patientenkontakt in Deutschland („Online Hautarzt – AppDoc“)

**DOI:** 10.1007/s00105-020-04660-w

**Published:** 2020-07-29

**Authors:** Wiebke Sondermann, Christof von Kalle, Jochen S. Utikal, Dirk Schadendorf, Stefan Esser, Benjamin Durani, Hendrike Durani, Martin Jansen, Titus J. Brinker

**Affiliations:** 1 Klinik für Dermatologie, Venerologie und Allergologie, Universitätsklinikum Essen, Universität Duisburg-Essen, Hufelandstr. 55, 45122 Essen, Deutschland; 2 Nationales Centrum für Tumorerkrankungen (NCT), Deutsches Krebsforschungszentrum (DKFZ), 69120 Heidelberg, Deutschland; 3 BIH-Berlin Institute of Health, Abteilung: Klinisch-Translationale Wissenschaften, Charité Berlin, Berlin, Deutschland; 4 Klinische Kooperationseinheit Dermato-Onkologie, Deutsches Krebsforschungszentrum (DKFZ), 69120 Heidelberg, Deutschland; 5 Klinik für Dermatologie, Universitätsklinikum Mannheim, Universität Heidelberg, Mannheim, Deutschland; 6 Westdeutsches Tumorzentrum, Universitätsklinikum Essen, Universität Duisburg-Essen und Deutsches Konsortium für Translationale Krebsforschung (DKTK), Essen, Deutschland; 7 Hautarztpraxis Dres. Durani, Bergheimer Str. 56a, 69115 Heidelberg, Deutschland; 8 Hautarztpraxis Dr. Martin Jansen, Bismarckstr. 5, 69115 Heidelberg, Deutschland

**Keywords:** Fernbehandlung, Qualitätssicherung, Teledermatologische Triage, Fachärztliche Begutachtung, Zweitbegutachtung, Remote treatment, Quality assurance, Teledermatological triage, Specialist assessment, Second opinion

## Abstract

**Hintergrund:**

Die Teledermatologie adressiert das Problem des Fachärztemangels und der oft langen Wartezeit auf einen Termin beim Dermatologen. Das Modellprojekt „Online Hautarzt – AppDoc“ ermöglicht eine schnelle anonyme fachärztliche Begutachtung und wurde am 22.10.2018 von der Landesärztekammer Baden-Württemberg als Modellprojekt für 2 Jahre genehmigt.

**Ziel der Arbeit (Fragestellung):**

Das Ziel der vorliegenden Arbeit ist die Präsentation der ersten realen Versorgungsdaten für die deutsche Teledermatologie im Rahmen der externen Qualitätssicherung des Modellprojektes „Online Hautarzt – AppDoc“.

**Material und Methoden:**

Anonyme Datensätze, die zwischen dem 21.11.2018 und 01.08.2019 bei „Online Hautarzt – AppDoc“ eingesendet wurden, wurden an der Universitäts-Hautklinik Essen qualitativ und quantitativ analysiert. Zusätzlich zur Auswertung der bislang eingesendeten Datensätze wurden 100 eingesandte Fälle fachärztlich zweitbegutachtet.

**Ergebnisse:**

Insgesamt flossen 1364 Fälle (60,4 % Männer, 39,6 % Frauen) in die jetzige erste externe wissenschaftliche Evaluation ein. In 90,3 % der Fälle war die Stellung einer Ferndiagnose möglich. Die beiden häufigsten Diagnosen waren verschiedene Ekzemformen (*n* = 270) und Nävi (*n* = 163). Fast zwei Drittel der Patienten (64,3 %) konnten rein teledermatologisch behandelt werden. Die stichprobenartige Zweitbegutachtung von 100 Fällen ergab eine Übereinstimmung der Diagnose unter Einbeziehung der Differenzialdiagnose(n) in 97 % der Fälle.

**Diskussion:**

Die erste externe wissenschaftliche Evaluation des Modellprojektes** „**Online Hautarzt – AppDoc“ ergab, dass die Reduktion von räumlichen und zeitlichen Barrieren einer hautfachärztlichen Begutachtung sowie die teledermatologische Triage bislang gelungen sind.

## Abstract (en)

**Background:**

Teledermatology addresses the problems associated with the lack of specialists and the often long waiting time for an appointment with a dermatologist. The research project Online Dermatologist—AppDoc enables a fast anonymous expert opinion and was approved on 22 October 2018 by the Landesärztekammer Baden-Württemberg for 2 years as a model project.

**Objectives:**

The aim of the present work is the presentation of the first real healthcare data for German teledematology within the framework of the external quality assurance of the model project Online Dermatologist—AppDoc.

**Materials and methods:**

Anonymous data records submitted to Online Dermatologist—AppDoc between 21 November 2018 and 1 August 2019 were analyzed qualitatively and quantitatively at the Department of Dermatology of the University Hospital Essen. In addition to the evaluation of the data records submitted so far, 100 cases submitted underwent a second assessment by a board-certified dermatologist to assess concordance.

**Results:**

A total of 1364 cases (60.4% men, 39.6% women) were included in the current first external scientific evaluation. In 90.3% of the cases, remote diagnosis was possible. The two most frequent diagnoses were different forms of eczema (*n* = 270) and nevi (*n* = 163). Almost two thirds of the patients (64.3%) could be treated teledermatologically only. The random second examination of 100 cases resulted in an agreement of the diagnosis including the differential diagnosis/diagnoses in 97% of the cases.

**Conclusions:**

The first external scientific evaluation of the teledermatological model project Online Dermatologist—AppDoc indicates that the reduction of spatial and temporal barriers of a dermatological examination as well as the teledermatological triage have been so far successful.

## Hintergrund und Fragestellung

In Deutschland weisen etwa 25 % der erwachsenen Bevölkerung jährlich einen dermatologischen Behandlungsbedarf auf [1]. Je nach Region warten Patienten oft mehrere Monate auf eine fachärztliche Ersteinschätzung ihres Hautproblems. In dieser Zeit können sich Hautkrankheiten von dem Stadium einer kurativ behandelbaren Krankheit zu einer dauerhaften Gesundheitseinschränkung entwickeln. Demgegenüber sind viele Hautarztbesuche nicht unbedingt erforderlich und könnten über eine teledermatologische Triage reduziert werden. Bislang waren teledermatologische Ansätze in Deutschland nicht weit verbreitet [2].

Die Pandemie mit SARS-CoV‑2 stellt momentan einen Treiber für telemedizinische Applikationen dar [12]. Bereits seit Februar 2020 führt beispielsweise die Techniker Krankenkasse ein telemedizinisches Pilotprojekt („TK-Onlinesprechstunde“) zu 8 Symptomen/Krankheitsbildern durch, das erstmals erfolgreich eine komplette Behandlungskette von der Diagnose über die Krankschreibung bis hin zur Medikamentenbestellung in einem durchgängig digitalen Prozess abbildet [11]. Die Begrenzungsregelung bei Videosprechstunden wurde durch die Kassenärztliche Bundesvereinigung (KBV) und den Spitzenverband der gesetzlichen Krankenkassen für das zweite Quartal 2020 aufgehoben. Vorher durften Ärzte maximal 20 % der Behandlungsfälle mittels Videosprechstunde behandeln [10]. Bisherige telemedizinische Ansätze in Deutschland bedurften zudem der persönlichen Bekanntheit von Arzt und Patient (Fernbehandlungsverbot) [2]. Im Rahmen der aus der Pandemie mit SARS-CoV‑2 resultierenden Regelung ist es nun auch möglich, die Videosprechstunde einzusetzen, wenn ein Patient vorher noch nicht in der Praxis war [10]. Hierbei handelt es sich definitiv um signifikante Fortschritte in der Anwendbarkeit der Telemedizin, jedoch muss einschränkend erwähnt werden, dass die Technologie der Videotelefonie die Arbeitslast von Ärztinnen und Ärzten potenziell eher erhöht als vermindert, möglicherweise sogar kostenintensiver ist und einer schnellen Internetverbindung bedarf, die in ländlichen Regionen keine Selbstverständlichkeit darstellt [6, 14].

Das Modellprojekt „Online Hautarzt – AppDoc“ wurde in kooperativer Zusammenarbeit von Ärzten und Wissenschaftlern des Nationalen Centrums für Tumorerkrankungen, der Universitäts-Hautklinik Heidelberg und niedergelassenen Hautfachärzten konzipiert und durch das lokale Technologieunternehmen Smart Health Heidelberg GmbH umgesetzt. Es handelt sich um eine Store-and-Forward-Technologie, da die Befunde zwischenzeitlich gespeichert und zeitlich versetzt bearbeitet werden. Die Store-and-Forward-Teledermatologie bietet verschiedene Vorteile gegenüber der Live-Videodermatologie. So ist beispielsweise weder eine schnelle Internetverbindung noch eine Terminvereinbarung zwischen Arzt und Patient erforderlich [6].

Das Ziel des Modellprojektes „Online Hautarzt – AppDoc“ besteht neben der Reduktion von räumlichen sowie zeitlichen Barrieren darin, bei Haut- und Geschlechtskrankheiten eine anonyme fachärztliche Begutachtung zu ermöglichen, die die Privatsphäre des Patienten akzeptiert und auch aus Datenschutzsicht eine sichere Alternative zu bisherigen Ansätzen darstellt.

Das Modellprojekt „Online Hautarzt – AppDoc“ wurde am 22.10.2018 von der Landesärztekammer Baden-Württemberg für 2 Jahre genehmigt und am 21.11.2018 mit flankierenden Pressemitteilungen der Universitätsmedizin Essen und des Nationalen Centrums für Tumorerkrankungen in Heidelberg und des Universitätsklinikums Heidelberg online geschaltet.

### Ablauf der digitalen Behandlung aus Patientensicht

**Figure 1:**
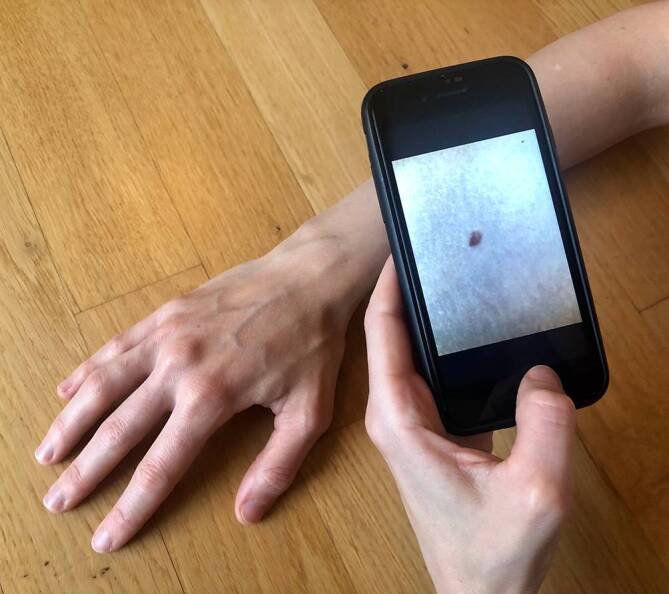

Der Patient kann „AppDoc“ über den Browser (https://online-hautarzt.net) oder über die Apps für Android und iPhones benutzen. Beim ersten Öffnen der App wird ein interessierter Patient mit einem Hautproblem über Datenschutz, Limitationen von Ferndiagnostik (v. a. Verschreibung von Medikamenten/Notfälle) aufgeklärt. Ist der Patient einverstanden, kann er sein Hautproblem an einen Hautfacharzt senden. Dazu beantwortet er wenige Fragen (Alter, Geschlecht, Beschwerden, einseitiges/beidseitiges Auftreten der Beschwerden, Lokalisation, bisherige Behandlungsversuche, weitere, optionale Informationen). Angegeben werden müssen: Alter, Geschlecht sowie Dauer der Beschwerden. Außerdem wird der Patient dazu aufgefordert 1 Übersichtsaufnahme (ca. 30 cm Abstand) von der betroffenen Hautstelle (Handykamera oder gespeichertes Bild auf dem Smartphone) sowie 2 Nahaufnahmen aus unterschiedlichen Blickwinkeln (10 cm Abstand) zur besseren Beurteilung des räumlichen Aspekts der Hautläsion (Abb. 1) bereitzustellen. In einem Freitextfeld soll abschließend das Hautproblem genauer beschrieben werden. Alle anderen Angaben, auch die Angabe einer E‑Mail-Adresse, sind optional. Dem Patienten wird bei der Erstellung seines Falles geraten, keine identifizierenden Informationen/Bilder hochzuladen, um einen anonymen Datensatz zu gewährleisten. Anschließend wird der Patient zur sicheren Zahlung des Service aufgefordert (nach GOÄ), die entkoppelt von seinen Falldaten über einen externen Zahlungsanbieter durchgeführt wird. Für die Bearbeitung des Falles werden 24,95 € erhoben. Eine vorherige Preisstaffelung je nach Befundungszeit (8–48 h) entfiel Mitte 2019. Nach der Zahlung erfolgt die Übertragung der Daten über eine verschlüsselte Verbindung auf einen gesicherten Server in Deutschland. Der Patient erhält eine anonyme Zahlen-Buchstaben-Kombination, über die er auf seinen individuellen, verschlüsselten Datenraum sicher zugreifen kann (sowohl über die App als auch über die Webseite www.online-hautarzt.net). Patienten können ihren Fall bearbeiten und anpassen, solange dieser noch nicht abgesendet ist. Falls es bis zum Erhalt der Empfehlung zu einer akuten Änderung des Hautbefundes gekommen sein sollte, könnte dem befundenen Dermatologen nach der digitalen Behandlung des Falles über die Rückfragemöglichkeit eine Änderung des Befundes mitgeteilt werden. Die Bearbeitung erfolgt innerhalb von 24 h; im Durchschnitt sogar in knapp unter 30 min. Wurde der Fall bearbeitet, erhält der Patient eine Push-Benachrichtigung auf sein Handy und zusätzlich eine E‑Mail, falls er seine Adresse angegeben hat (optional).

### Ablauf der digitalen Behandlung aus Arztsicht

Die Befundung erfolgt über ein verschlüsseltes Ärzteportal, auf das der Arzt von überall auf der Welt und zu jeder Zeit zugreifen kann, sofern er eine Internetverbindung hat. Befundende Hautfachärzte mit mindestens 10 Jahren Berufserfahrung aus Heidelberg erhalten eine E‑Mail, sobald ein neuer Fall eingegangen ist. Sobald ein Hautfacharzt begonnen hat, einen Fall zu bearbeiten, ist dieser für andere Kollegen gesperrt. Das Portal lässt zu, dass dem Patienten Rückfragen gestellt werden können und der Patient hierauf jeweils einmalig antworten kann. Am Ende der digitalen Behandlung füllt der behandelnde Hautfacharzt innerhalb der App einen digitalen Fragebogen zur erfolgten Behandlung aus, der der kontinuierlichen qualitativen und quantitativen Evaluation des Befundungs- und Behandlungsprozesses dient.

### Datenschutz

„AppDoc“ wurde im Rahmen der Datenschutz-Folgenabschätzung bewusst nach dem Prinzip der Datensparsamkeit und Datenminimierung der Datenschutzgrundverordnung (DSGVO Art. 5/1c) gestaltet, um Patienten eine weitestgehend anonyme Nutzung zu ermöglichen, die auch bei einem erfolgreichen Hackerangriff einen möglichen Schaden für Betroffene stark reduziert. Sämtliche Datenübertragungen erfolgen verschlüsselt, die Datenbank selber ist verschlüsselt, und die Fallnummer besteht aus einer 20-stelligen Zahlen-Buchstaben-Kombination, die auch durch Brute-force-Attacken (automatisiertes Erraten von Fall-Kennungen) über IP-Blockaden und Zugriffsbeschränkungen nicht erraten werden kann. Art. 5/1c der EU-DSGVO wurde als oberstes Prinzip des Datenschutzkonzeptes von „AppDoc“ gewählt, da in jüngerer Vergangenheit wiederholt demonstriert werden konnte, dass Telemedizindienstleister in Deutschland und Europa trotz des Einsatzes von moderner Verschlüsselungstechnologie weder erfolgreich noch nachhaltig vor Hackerangriffen geschützt werden konnten [18].

### Qualitätskontrolle

Neben der sorgfältigen Auswahl und Schulung der behandelnden Hautfachärzte (Dr. Benjamin Durani, Dr. Hendrike Durani und Dr. Martin Jansen aus Heidelberg), wie im aktuellen Leitfaden zur Praxis der Teledermatologie der deutschsprachigen Dermatologen empfohlen [2], sollen anonyme Datensätze für qualitative und quantitative Analysen genutzt werden, um den Befundungsprozess für Ärzte und Patienten weiter zu optimieren. Die Evaluation erfolgt zum einen über den lokalen wissenschaftlichen Beirat, bestehend aus Ärzten und Wissenschaftlern des Nationalen Centrums für Tumorerkrankungen sowie der Universitäts-Hautklinik Heidelberg, sowie lokaler Hautarztpraxen. Zum anderen erfolgt eine regelmäßige (mindestens 1‑mal jährliche) unabhängige, externe wissenschaftliche Evaluation des Modellprojektes an der Universitäts-Hautklinik Essen (Leitung: Frau Dr. Wiebke Sondermann), deren Ergebnisse nun vorgestellt werden sollen.

## Studiendesign und Untersuchungsmethoden

### Externe wissenschaftliche Evaluation

Im Rahmen der externen wissenschaftlichen Evaluation an der Universitäts-Hautklinik Essen wurden anonyme Datensätze aus „AppDoc“ qualitativ und quantitativ mit dem Ziel der Sicherung der Qualität analysiert.

Die externe wissenschaftliche Evaluation unter der Leitung der Fachärztin für Haut- und Geschlechtskrankheiten, Frau Dr. Wiebke Sondermann, wurde von der Landesärztekammer Baden-Württemberg im Rahmen des Modellprojektes zum Zwecke der Qualitätssicherung genehmigt.

Für die aktuell erste externe wissenschaftliche Evaluation wurden einerseits alle eingesandten Fälle von der Freischaltung der Applikation „Online Hautarzt – AppDoc“ am 21.11.2018 bis zum 01.08.2019 statistisch-deskriptiv ausgewertet. Die anonymen Patientendatensätze wurden Frau Dr. Wiebke Sondermann hierfür am 17.09.2019 über einen verschlüsselten Kanal in tabellarischer Form zur Verfügung gestellt. Darüber hinaus erfolgte im Rahmen der qualitativen Evaluation eine stichprobenartige Zweitbefundung von eingesandten anonymen Patientenfällen durch die Leiterin der externen wissenschaftlichen Evaluation, Frau Dr. Wiebke Sondermann.

Die Patientendatensätze enthielten die folgenden Informationen:

- drei Fotos der Hautveränderung (aus 30 cm Entfernung und 2 Bilder aus 10 cm Entfernung aus einem jeweils anderen Blickwinkel),
- Alter und Geschlecht des Patienten,
- aktuelle Beschwerden (Auswahl zwischen Ausschlag, Juckreiz, Schwellung, Rötung, Schmerzen, Schuppung, Muttermal, Flecken, Sonstiges),
- einseitiges/beidseitiges Auftreten der Beschwerden bzw. nicht sicher,
- die von den Hautveränderungen betroffene Körperregion,
- Dauer der Beschwerden (Auswahl zwischen weniger als 2 Tage, zwischen 2 bis 6 Tagen, zwischen 1 bis 4 Wochen, länger als 1 Monat, chronisch/permanent, andere Angabe),
- Vortherapie ja/nein,
- weitere Informationen als Freitext,
- fakultative Bewertung der App am Ende des Behandlungsprozesses auf einer Skala von 1 bis 5 Sternen (5 Sterne entspricht der bestmöglichen Bewertung) sowie in Form von Freitextkommentaren.

Um den Befundungs- und Behandlungsprozesses kontinuierlich qualitativ und quantitativ evaluieren zu können, ist, wie oben bereits angesprochen, in der App die Erfassung von Angaben zur erfolgten Befundung und Behandlung durch den Arzt implementiert. Nach jedem Befundungs- und Behandlungsprozess füllt der behandelnde Arzt innerhalb der App einen digitalen Fragebogen aus, mit dessen Hilfe folgende Angaben erhoben werden:

- ob die Stellung einer Diagnose möglich war, und wenn ja, um welche Diagnose es sich handelte,
- ob ein Arztbesuch beim Dermatologen vor Ort erforderlich war und dementsprechend empfohlen wurde,
- ob eine Behandlung empfohlen wurde und, wenn ja, welche.

Die statistisch-deskriptive Auswertung erfolgte mit dem Programm SPSS (Statistical Package for the Social Sciences, IBM Inc., Chicago, Illinois) Version 25.

Folgende Angaben wurden in die Auswertung mit einbezogen:

- Alter und Geschlecht des Patienten,
- über welches Medium der Fall eingesandt worden war (iOS, Android, Internet),
- ob eine Vortherapie erfolgt war,
- ob die Stellung einer Diagnose möglich war,
- Art der Diagnose(n),
- ob ein Arztbesuch beim Dermatologen vor Ort erforderlich war und dementsprechend empfohlen wurde,
- ob eine Behandlung empfohlen wurde,
- Art der empfohlenen Behandlung(en),
- Bewertung durch den Patienten.

Für die stichprobenartige Zweitbefundung von eingesandten anonymen Patientenfällen verfügte Frau Dr. Wiebke Sondermann über einen temporären Zugang zur Plattform des „Online Hautarztes – AppDoc“. Personenbezogene Daten waren für sie nicht sichtbar. Im Rahmen der aktuellen Evaluation wurden zur Überprüfung der Qualität stichprobenartig 100 eingesandte Fälle fachärztlich zweitbegutachtet.

Folgende Aspekte wurden in diese Auswertung mit einbezogen:

- ob die Diagnose des „AppDoc“-Befunders mit der des Zweitbefunders übereinstimmte bzw. dass Übereinstimmung darin bestand, dass keine Ferndiagnose möglich war,
- wenn nein, inwiefern die Differenzialdiagnose übereinstimmte,
- ob sich der Patient laut des „AppDoc“-Befunders beim Hautarzt vor Ort vorstellen sollte,
- ob sich der Patient laut des Zweitbefunders beim Hautarzt vor Ort vorstellen sollte,
- wie oft die Einschätzung einer erforderlichen Vorstellung beim Hautarzt vor Ort übereinstimmte,
- ob die empfohlene Therapie des „AppDoc“-Befunders mit der des Zweitbefunders übereinstimmte oder zumindest partiell übereinstimmte.

## Ergebnisse

### Auswertung der eingesendeten Fälle

Insgesamt flossen 1364 Fälle in die jetzige erste externe wissenschaftliche Evaluation ein.

Mehr Männer (*n* = 824, 60,4 %) als Frauen (*n* = 540, 39,6 %) nutzten die Anwendung, um ihr Hautproblem teledermatologisch behandeln zu lassen. Das mittlere Alter der Patienten betrug 42,52 ± 16 Jahre bei einer Spanne von <1 Jahr bis 96 Jahren.

Etwas mehr als die Hälfte der Patienten (*n* = 786, 57,6 %) sandte ihren Fall über ein mobiles Endgerät ein (32,7 % über iOS und 24,9 % über Android), die restlichen Fälle wurden über die Homepage eingeschickt (*n* = 578, 42,4 %).

Mehr als die Hälfte der Patienten (*n* = 832, 61 %) hatten bisher keinerlei Vortherapie angewendet gegenüber *n* = 532 (39 %) Patienten, die bereits eine Vorbehandlung erhalten hatten.

Die Auswertung des digitalen Ärztefragebogens zeigte, dass in 90,3 % (*n* = 1232) der Fälle laut der befundenden Ärzte die Stellung einer Ferndiagnose möglich war.

Die Analyse der gestellten Diagnosen ergab ein sehr breites Bild an eingesandten Hautproblemen. Es wurden über 80 verschiedene Diagnosen gestellt (vgl. Tab. 1). Bei 24 Patienten wurden 2 Diagnosen gestellt, sodass insgesamt 1256 Diagnosen bei 1364 Einsendungen gestellt wurden.

**Table 1:** 

	Häufigkeit (*n*)	Prozent an allen Diagnosen	Prozent an allen Fällen
Ekzem (verschiedene Formen)	270	21,5	19,8
Nävus	163	13,0	12,0
Seborrhoische Keratose	71	5,6	5,2
Warze/Akanthom	54	4,3	4,0
NMSC inklusive Vorstufen	52	4,1	3,8
Exanthem	41	3,3	3,0
Gefäßveränderungen (u. a. Angiom)	34	2,7	2,5
Herpes-Infektion	28	2,2	2,1
Iktusreaktion	25	2,0	1,8
Bakterielle Hautinfektion	25	2,0	1,8
Pityriasis versicolor	24	1,9	1,8
Hämatom	21	1,7	1,5
Akne	21	1,7	1,5
Mykose	20	1,6	1,5
Follikulitis	20	1,6	1,5
Fibrom	20	1,6	1,5
Kondylome	17	1,4	1,2
Pityriasis rosea	17	1,4	1,2
Urtikaria	16	1,3	1,2
Balanitis	16	1,3	1,2
Psoriasis	16	1,3	1,2
Lentigo	15	1,2	1,1
Rosazea	15	1,2	1,1
Pigmentstörung	14	1,1	1,0
Zyste	14	1,1	1,0
Intertrigo	12	1,0	0,9
Purpura pigmentosa progressiva	11	0,9	0,8
AD (inklusive Minimalvarianten)	11	0,9	0,8
Nagelveränderungen	10	0,8	0,7
Granuloma anulare	10	0,8	0,7
Sonstige Diagnose	9	0,7	0,7
Lichen ruber planus	9	0,7	0,7
Mollusken	8	0,6	0,6
LSA	7	0,6	0,5
Skabies	6	0,5	0,4
Abszess	6	0,5	0,4
Periorale Dermatitis	6	0,5	0,4
Talgdrüsenhyperplasie	6	0,5	0,4
Atherom	6	0,5	0,4
Narbe	6	0,5	0,4
Borreliose	6	0,5	0,4
Kontaktallergie	6	0,5	0,4
Granuloma pyogenicum	5	0,4	0,4
Sonstige Dermatitis	5	0,4	0,4
Wunde/Wundheilungsstörung	4	0,3	0,3
LE/„lymphocytic infiltration of the skin“	4	0,3	0,3
Lichtdermatose	4	0,3	0,3
Arzneireaktion	4	0,3	0,3
Vaskulitis	4	0,3	0,3
Stauungsdermatitis/-ekzem	4	0,3	0,3
Erythrosis interfollicularis colli	4	0,3	0,3
Prurigo	3	0,2	0,2
Keratosis follicularis	3	0,2	0,2
Riesenkomedo	3	0,2	0,2
Cheilitis	3	0,2	0,2
Hirsuties penis papillaris	3	0,2	0,2
Porokeratose	3	0,2	0,2
Lues	2	0,2	0,1
Morbus Grover	2	0,2	0,1
Gutartiger Hauttumor	2	0,2	0,1
Xerosis cutis	2	0,2	0,1
Vitiligo	2	0,2	0,1
Erythema nodosum	2	0,2	0,1
Alopezie	2	0,2	0,1
Kongenitale Hautanomalie	2	0,2	0,1
Trombidiose	2	0,2	0,1
Lichen vidal	2	0,2	0,1
Phimose	2	0,2	0,1
Veränderungen der Zunge	2	0,2	0,1
Hyperkeratose	2	0,2	0,1
Parapsoriasis	1	0,1	0,1
Larva migrans	1	0,1	0,1
Necrobiosis lipoidica	1	0,1	0,1
Acanthosis nigricans	1	0,1	0,1
Analfissur	1	0,1	0,1
PUPP	1	0,1	0,1
MM	1	0,1	0,1
Aphthe	1	0,1	0,1
Sarkoidose	1	0,1	0,1
Acne inversa	1	0,1	0,1

*NMSC* „non-melanoma skin cancer“, *AD* atopische Dermatitis, *LE* Lupus erythematodes, *LSA* Lichen sclerosus et atrophicans, *PUPP* „pruritic urticarial papules and plaques of pregnancy“, *MM* malignes Melanom

**Figure 2:**
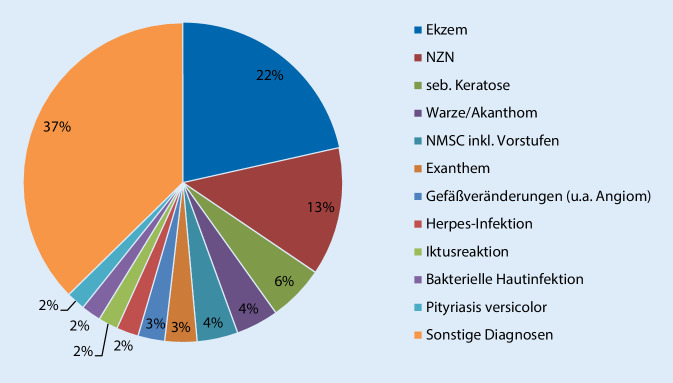

Die beiden häufigsten Diagnosen waren mit Abstand verschiedene Ekzemformen (*n* = 270, 21,5 % an allen Diagnosen bzw. 19,8 % an allen Einsendungen) und Nävi (*n* = 163, 13 % an allen Diagnosen bzw. 12 % an allen Einsendungen). An dritter Stelle folgten seborrhoische Keratosen (*n* = 71, 5,6 % an allen Diagnosen bzw. 5,2 % an allen Einsendungen). Einen Überblick über die häufigsten Diagnosen (>21 Fälle) an allen Diagnosen gibt Abb. 2.

Die weitere Auswertung des digitalen Ärztefragebogens zeigte, dass gut einem Drittel der Patienten (*n* = 487, 35,7 %) empfohlen wurde, sich beim Hautfacharzt vor Ort vorzustellen. Die restlichen knapp zwei Drittel der Patienten (*n* = 877, 64,3 %) konnten, zumindest zum Zeitpunkt der Einsendung des Falles bei „Online-Hautarzt App-Doc“, rein teledermatologisch behandelt werden.

Fast die Hälfte der Patienten (*n* = 640, 46,9 %) erhielt eine medikamentöse Therapieempfehlung.

Insgesamt wurden bei 148 Patienten 2 Therapieempfehlungen ausgesprochen, 26 Patienten erhielten 3 verschiedene Therapieempfehlungen, und 4 Patienten wurden sogar 4 verschiedene Therapieempfehlungen ausgesprochen.

**Figure 3:**
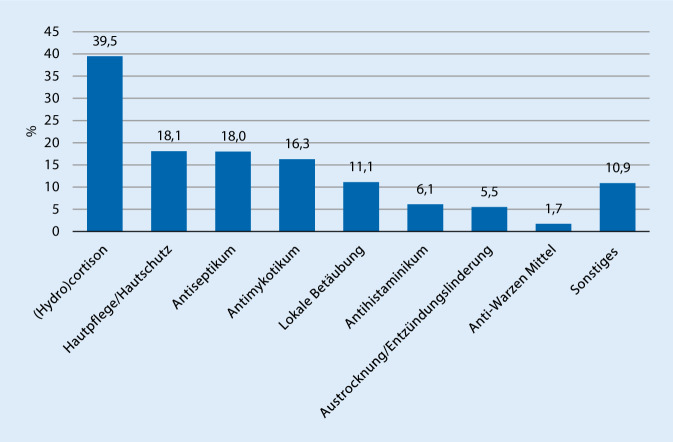

Die Therapieempfehlungen ließen sich über 20 verschiedenen Therapieansätzen zuordnen. Die 4 am häufigsten empfohlenen Therapieansätze waren (Hydro)cortisonhaltige Externa in insgesamt 253 Fällen (18,5 % an allen Fällen bzw. 39,5 % an allen Fällen, in denen eine Therapieempfehlung gegeben wurde), Hautpflege bzw. Hautschutzpräparate in 116 Fällen (8,5 %/18,1 %), Antiseptika in 115 Fällen (8,4 %/18 %) sowie Antimykotika bei 104 Patienten (7,6 %/16,3 %) (Abb. 3.).

In maximal 40 % der Fälle waren Rückfragen an den Patienten erforderlich. Diese betrafen zumeist ungenaue Angaben zur Eigenanamnese, den Vorbehandlungen und den aktuellen Beschwerden. Bei ca. 10–20 % der Rückfragen wurden verbesserte Bilder angefordert.

Etwa 38 % der Patienten begannen, einen Fall zu erstellen, und schlossen diesen dann nicht ab, zumeist unmittelbar vor dem Bezahlvorgang.

Insgesamt 300 Patienten gaben am Ende des teledermatologischen Behandlungsprozesses eine Bewertung in Form von „Sternen“ ab. Der Durchschnittswert der Bewertungen betrug 4,5 Sterne, und der größte Anteil der Bewertungen entfiel auf die beste Bewertung „5 Sterne“ (*n* = 231, 77 % an allen Bewertungen). Es wurden insgesamt 374 Freitextkommentare abgegeben, von denen 344 (92 %) positiv ausfielen und 30 (8 %) einen kritischen bis negativen Charakter hatten.

### Auswertung der stichprobenartig zweitbegutachteten Fälle

Die Auswertung der 100 stichprobenartig zweitbegutachteten Fälle ergab, dass in 80 % der Fälle die primäre Verdachtsdiagnose mit der des „AppDoc“-Befunders übereinstimmte bzw. dass Übereinstimmung darin bestand, dass keine Ferndiagnose möglich war. Unter Hinzuziehung der Differenzialdiagnose(n) stieg die Übereinstimmung auf 97 %. In Tab. 2 finden sich alle Diagnosen, bei denen der „App-Doc“-Befunder und der Zweitgutachter übereinstimmten. Tabelle 3 bietet eine Übersicht über die differierenden Diagnosen. Zudem ist ersichtlich, ob der „AppDoc“-Befunder bzw. der Zweitgutachter eine Vorstellung beim niedergelassenen Hautfacharzt empfahl. In 4 Fällen, in den die Verdachtsdiagnose nicht übereinstimmte und die Zweitbefundung eine Vorstellung beim Hautfacharzt empfahl, lautete die Empfehlung des „AppDoc“-Befunders, sich bei fehlender Besserung bzw. Veränderung der Hautveränderung beim Hautarzt vor Ort vorzustellen. Insgesamt erhielten unter den 100 stichprobenartig zweitbegutachteten Fällen 53 Patienten seitens des „AppDoc“-Befunders die Empfehlung, beim Hautfacharzt vorstellig zu werden. Genauso vielen Patienten hätte der Zweitgutachter den Besuch beim Hautfacharzt vor Ort empfohlen. In 19 Fällen differierte die Meinung des Zweitgutachters und des „AppDoc“-Befunders, ob dem Patienten der Besuch des Hautfacharztes vor Ort empfohlen werden sollte.

**Table 2:** 

	Häufigkeit (*n*)	Prozent an allen übereinstimmenden Diagnosen
Herpes-Infektion	6	8,7
Nävus	5	7,2
Bakterielle Hautinfektion	4	5,8
Kondylome	4	5,8
Balanitis	3	4,3
LSA	2	2,9
Ekzem	2	2,9
Lentigo	2	2,9
Prurigo	2	2,9
Follikulitis	2	2,9
AD (inklusive Minimalvarianten)	2	2,9
Abszess	2	2,9
Arzneireaktion	2	2,9
Stauungsdermatitis/-ekzem	2	2,9
Mollusken	2	2,9
Vaskulitis	2	2,9
NMSC inklusive Vorstufe	1	1,4
Pityriasis versicolor	1	1,4
Urtikaria	1	1,4
Hämatom	1	1,4
Exanthem	1	1,4
Seborrhoische Keratose	1	1,4
Pigmentstörung	1	1,4
Riesenkomedo	1	1,4
Angiom	1	1,4
Psoriasis	1	1,4
Skabies	1	1,4
Wunde/Wundheilungsstörung	1	1,4
Acne inversa	1	1,4
Rosazea	1	1,4
Nagelveränderungen	1	1,4
Erythema nodosum	1	1,4
Alopezie	1	1,4
Granuloma anulare	1	1,4
LE/„lymphocytic infiltration of the skin“	1	1,4
Borreliose	1	1,4
Fibrom	1	1,4
Trombidiose	1	1,4
Aphthe	1	1,4
Normalbefund	1	1,4
Hyperkeratose	1	1,4

*NMSC* „non-melanoma skin cancer“, *AD* atopische Dermatitis, *LE* Lupus erythematodes, *LSA* Lichen sclerosus et atrophicans

**Table 3:** 

„AppDoc“	Zweitbefunder	„AppDoc“	Zweitbefunder
Diagnose	Diagnose	Vorstellung HFA empfohlen	Vorstellung HFA empfohlen
Seborrhoische Keratose	Nävus	Ja	Ja
Pityriasis rosea	Ekzem	Ja	Ja
Warze/Akanthom	Seborrhoische Keratose	Nein	Nein
Ekzem	Lichen ruber	Nein	Ja
Pilzinfektion	Ekzem	Nein	Nein
Skabies	Ekzem	Nein	Nein
Ekzem	Pilzinfektion	Nein	Nein
Ekzem	Lichen vidal	Nein	Nein
Mollusken	Warze/Akanthom	Nein	Nein
Pilzinfektion	Psoriasis	Nein	Ja
Pilzinfektion	Ekzem	Nein	Nein
Kontaktallergie	Sonstige Dermatitis	Ja	Nein
Urtikaria	Exanthem	Nein	Ja
Ekzem	Pilzinfektion	Nein	Nein
Pityriasis rosea	Keine sichere Ferndiagnose möglich	Nein	Nein
Pityriasis rosea	Psoriasis	Nein	Nein
Keine sichere Ferndiagnose möglich	Warze/Akanthom	Ja	Nein
Angiom	Keine sichere Ferndiagnose möglich	Nein	Ja
Seborrhoische Keratose	Keine sichere Ferndiagnose möglich	Nein	Ja
Nävus	Keine sichere Ferndiagnose möglich	Ja	Ja

*HFA* Hautfacharzt

Insgesamt 44 der 100 Patienten wurde eine freiverkäufliche Therapie empfohlen. Die empfohlene Therapie (sofern eine Therapie empfohlen wurde) stimmte in 45,5 % der Fälle genau zwischen der des „AppDoc“-Befunders und der des Zweitgutachters überein. Unter Hinzuzählung der partiellen Übereinstimmung des empfohlenen Therapieregimes stieg dieser Wert auf 72,8 %. Tabelle 4 stellt die differierenden Therapieempfehlungen dar, die sich zum Teil aus unterschiedlichen Verdachtsdiagnosen ergaben; teilweise wurde bei derselben vermuteten Verdachtsdiagnose eine andere Therapieempfehlung ausgegeben. Es fanden sich keine Differenzen, bei denen man mit gravierenden negativen Konsequenzen für den Patienten rechnen würde.

**Table 4:** 

„AppDoc“		Zweitbefunder	
Diagnose	Therapie	Diagnose	Therapie
Pigmentstörung	Depigmentierendes Externum	Pigmentstörung	Lichtschutz
Ekzem	(Hydro)cortison	Lichen ruber	Rezeptpflichtiges wurde empfohlen
Pilzinfektion	Antimykotikum	Ekzem	(Hydro)cortison
Ekzem	(Hydro)cortison	Pilzinfektion	Antimykotikum
Mollusken	Mittel gegen Mollusken	Warze/Akanthom	Anti-Warzen-Medikament
Pilzinfektion	Antimykotikum	Psoriasis	Rezeptpflichtiges wurde empfohlen
Urtikaria	Antihistaminikum	Exanthem	Rezeptpflichtiges wurde empfohlen
Herpes-Infektion	Austrocknendes/entzündungslinderndes Externum	Herpes-Infektion	Antiseptikum
Rosazea	Rezeptpflichtiges wurde empfohlen	Rosazea	Hautpflege/Hautschutz und Lichtschutz
Pigmentstörung	Depigmentierendes Externum	Pigmentstörung	Lichtschutz
Abszess	Zugsalbe und Antiseptikum	Abszess	Rezeptpflichtiges wurde empfohlen
Keine sichere Ferndiagnose möglich	Keine Therapie empfohlen	Warze/Akanthom	Anti-Warzen-Medikament
Warze/Akanthom	Anti-Warzen-Medikament	Seborrhoische Keratose	„Watch and wait“

## Diskussion

Das Modellprojekt „Online Hautarzt – AppDoc“ ist die erste und bislang einzige unabhängig von der aktuellen Pandemiesituation genehmigte Lösung für teledermatologische Befundung ohne vorherigen Arztkontakt in Deutschland und wurde am 22.10.2018 von der Landesärztekammer Baden-Württemberg für 2 Jahre bewilligt. Vorangegangen waren zahlreiche Studien, die eine sehr gute diagnostische Präzision teledermatologischer Diagnosen zeigen konnten [4, 7, 9, 16]. Der aktuelle Leitfaden zur Praxis der Teledermatologie der deutschsprachigen Dermatologen [2] empfiehlt, den Einsatz teledermatologischer Verfahren immer dann in Erwägung zu ziehen, wenn ein relevanter Zusatznutzen für die Patienten ohne relevante Nachteile für sie und die Versorgenden zu erwarten ist. Um zu überprüfen, ob diese Voraussetzungen in der Realität gegeben sind, ist eine wissenschaftliche Evaluation (neuer) teledermatologischer Anwendungen unerlässlich. Der aktuelle Leitfaden zur Praxis der Teledermatologie der deutschsprachigen Dermatologen [2] empfiehlt zudem, dass sowohl die technischen Abläufe als auch die medizinische Qualität ständig kontrolliert werden. Medizinisch sollten die teledermatologischen Befunde stichprobenartig von externen Experten und Expertinnen überprüft werden, um so die teledermatologischen Anwendungen stetig verbessern zu können [2].

Entsprechend diesen Empfehlungen erfolgte nun unter der Leitung von Frau Dr. Wiebke Sondermann die erste externe wissenschaftliche Evaluation des von Heidelberger Ärzten und Wissenschaftlern initiierten Modellprojektes „Online Hautarzt – AppDoc“ an der Universitäts-Hautklinik Essen.

Es zeigte sich, dass in 90 % der Fälle die Stellung einer Ferndiagnose möglich war. Insgesamt wurden über 80 verschiedene Diagnosen gestellt. Die beiden häufigsten Diagnosen waren mit Abstand verschiedene Ekzemformen (19,8 % an allen Einsendungen) und Nävi (12 % an allen Einsendungen).

Die stichprobenartige, fachärztliche Zweitbefundung durch Frau Dr. Wiebke Sondermann ergab eine Übereinstimmung der primären Verdachtsdiagnose bzw. eine Übereinstimmung in der Tatsache, dass keine Ferndiagnose möglich war, mit der des „AppDoc“-Befunders (Hautfachärzte mit mindestens 10 Jahren Berufserfahrung) in 80 % der Fälle. Unter Hinzunahme der Differenzialdiagnose(n) stieg die Übereinstimmung auf 97 %. Es wurden keine Fälle identifiziert, die im Rahmen der Zweitbefundung als gravierende Fehldiagnosen eingeschätzt wurden. In 4 Fällen, in denen die Verdachtsdiagnose diskordant war und die Zweitbefundung eine Vorstellung beim Hautfacharzt empfohlen hätte, lautete die Empfehlung des „AppDoc“-Befunders, sich bei ausbleibender Besserung bzw. Veränderung der Hautveränderung beim Hautarzt vor Ort vorzustellen. Aus diesem Grund erscheinen signifikante negative Konsequenzen für den Patienten unwahrscheinlich.

Insgesamt 64,3 % der 1364 Patienten konnten zum Zeitpunkt der „AppDoc“-Konsultation rein teledermatologisch behandelt werden. Bei den 100 stichprobenartig zweitbefundeten Fällen betrug diese Quote sowohl für den „AppDoc“-Befunder als auch für den Zweitbegutachter allerdings nur 47 %. Fast die Hälfte der Patienten aus dem Gesamtkollektiv (47 %) und 44 % der Patienten aus der Zweitbegutachtung erhielten eine freiverkäufliche medikamentöse Therapieempfehlung. Die empfohlene Therapie stimmte in 45,5 % der Fälle genau und unter Hinzunahme einer teilweisen Übereinstimmung in 72,8 % der Fälle zwischen der des „AppDoc“-Befunders und der des Zweitgutachters überein. Es fanden sich unter den Therapieempfehlungen des „AppDoc“-Befunders und des Zweitbefunders keine Differenzen, bei denen man mit gravierenden negativen Konsequenzen für den Patienten rechnen würde.

Insgesamt sprechen die Daten für eine hohe diagnostische und therapeutische Qualität des „Online Hautarztes – AppDoc“. Die Bewertungen der Patienten waren, sofern eine Bewertung abgegeben worden war (22 % der Fälle), überwiegend sehr positiv.

Eine Limitation der Analyse besteht darin, dass es keinen aktiven Feedbackmechanismus für die Patienten außer der Bewertung mittels „Sternevergabe“ inklusive der Möglichkeit für einen Freitext gibt. Im Sinne der Qualitätsanalyse wäre ein standardisierter Follow-up-Mechanismus ideal, über den der Verlauf jedes einzelnen Patienten inklusive des Therapieansprechens dokumentiert und später analysiert werden könnte. Dies war und ist jedoch aufgrund von Sicherheitserwägungen (Datenschutz-Folgenabschätzung nach EU-DSGVO) nicht umsetzbar, da ein solches Follow-up die zusätzliche Erhebung von personenbezogenen Daten obligat macht. Man könnte jedoch darüber nachdenken, die Patienten bei der Einsendung unverbindlich zu bitten, nach beispielsweise 2 Wochen eine aktive Rückmeldung über den Verlauf zu geben und hierfür die technischen Möglichkeiten (beispielsweise ähnlich dem Fragebogen für die Befunder) zu schaffen. Hier wäre jedoch die Limitation gegeben, dass sich nur besonders motivierte und/oder sehr zufriedene bzw. sehr unzufriedene Patienten rückmelden könnten (Selektionsbias). Des Weiteren muss einschränkend erwähnt werden, dass der gewählte Studienaufbau keine Aussage darüber erlaubt, ob die telemedizinische Anwendung „Online Hautarzt – AppDoc“ im Vergleich zu einer Untersuchung und Behandlung beim Hautarzt vor Ort zu übereinstimmenden Ergebnissen kommt. Jedoch finden sich in der Literatur verschiedene Studien, die die diagnostische Präzision von teledermatologischen Ansätzen mit Face-to-Face-Konsultationen verglichen (sog. „Konkordanzstudien“). Ein Review aus dem Jahr 2016 ergab, dass die diagnostische Treffsicherheit bei Face-to-Face-Konsultationen in Bezug auf Hautkrebs im Vergleich zur Teledermatologie etwas besser war (67–85 % vs. 51–85 % Übereinstimmung mit dem Referenzstandard) [7]. Andere Studien konnten jedoch zeigen, dass die Teledermatologie, bezogen auf die diagnostische Präzision, vergleichbar mit einer Face-to-Face-Konsultation ist und teilweise sogar eine noch höhere diagnostische Treffsicherheit hat, was insbesondere auf die mittlerweile sehr hohe Auflösung von Handykameras zurückgeführt wird [3, 16, 19, 20].

Eine Möglichkeit, v. a. bei melanozytären Hautveränderungen die diagnostische Treffsicherheit bei teledermatologischer Behandlung zu erhöhen und Fehldiagnosen zu vermeiden, stellt die ergänzende Verwendung der Smartphone-Dermatoskopie dar [13, 17]. Weitere Ansätze, um den Anteil der Fehldiagnosen oder allgemeine Probleme bei der Anwendung von „Online Hautarzt – AppDoc“ oder anderen sog. Store-and-Forward-Technologien für Patient oder Arzt zu reduzieren, bestehen darin, dass sowohl Patient als auch Befunder dazu ermutigt werden sollten, großzügig von der Möglichkeit Gebrauch zu machen, bei Unklarheiten nachzufragen bzw. weitere Bilder anzufordern. Die Anwendung der App sollte so einfach wie möglich sein und einen höchstmöglichen Grad an Transparenz über die Abläufe bieten. Das Auftreten von technischen Problemen ist zu vermeiden.

## Ausblick

Teledermatologische Anwendungen werden im deutschen Versorgungssystem in den nächsten Jahren erheblich an Bedeutung gewinnen. Die Pandemie mit SARS-CoV‑2 hat der Teledermatologie wesentlichen Vorschub geleistet. Unabhängig von der aktuellen Pandemiesituation kann „Online-Hautarzt – AppDoc“ seit dem 08.04.2020 deutschlandweit angeboten werden und ist nicht mehr auf Baden-Württemberg beschränkt. Eine Übernahme der Kosten für teledermatologische Apps wie „AppDoc“ durch die Krankenkassen wäre wünschenswert, um Patienten nicht aus finanziellen Gründen von der Nutzung auszuschließen. Grundsätzlich scheint auch der assistierende Einsatz von künstlicher Intelligenz bei der Befundung von malignitätsverdächtigen Läsionen vielversprechend, muss jedoch zuvor in prospektiven Studien evaluiert werden [5, 15]. Aktuelle Daten weisen darauf hin, dass künstliche Intelligenz v. a. als Ergänzung, nicht aber als Ersatz den höchsten Mehrwert zu bieten scheint [8].

## Schlussfolgerung

Die erste externe wissenschaftliche Evaluation des teledermatologischen Modellprojektes „Online Hautarzt – AppDoc“ ergab, dass die Reduktion von räumlichen und zeitlichen Barrieren einer hautfachärztlichen Begutachtung sowie die teledermatologische Triage bislang im Sinne einer verbesserten Patientenversorgung gelungen sind. Zur Aufrechterhaltung und weiteren Verbesserung der Qualität der Anwendung sollten die Qualitätssicherungsprozesse weiterverfolgt werden.

## Fazit für die Praxis

- Die Teledermatologie adressiert das Problem des Fachärztemangels und der oft langen Wartezeit auf einen Termin beim Dermatologen.
- Das Modellprojekt „Online Hautarzt – AppDoc“ ermöglicht eine schnelle anonyme fachärztliche Begutachtung.
- Die erste externe wissenschaftliche Evaluation des Modellprojektes „Online Hautarzt – AppDoc“ ergab, dass die Reduktion von räumlichen und zeitlichen Barrieren einer hautfachärztlichen Begutachtung sowie die teledermatologische Triage bislang gelungen sind.
